# The Revalorization of Fishery By-Products: Types, Bioactive Compounds, and Food Applications

**DOI:** 10.1155/2024/6624083

**Published:** 2024-07-25

**Authors:** Diana Jimenez-Champi, Frank L. Romero-Orejon, Ana María Muñoz, Fernando Ramos-Escudero

**Affiliations:** ^1^ Nutrition Health Functional Foods and Nutraceuticals Research Unit Universidad San Ignacio de Loyola (UNUSAN-USIL), Lima, Peru; ^2^ Food Science and Nutrition Institute Universidad San Ignacio de Loyola (ICAN-USIL), Lima, Peru; ^3^ Health Sciences Faculty Universidad San Ignacio de Loyola, Lima, Peru

**Keywords:** bioactive compounds, fishery discards, fish protein isolate, food industry, functional properties

## Abstract

Recently, fish consumption has been increasing; subsequently, the number of by-products has also increased. However, generated residues are frequently discarded, and an appropriate management is necessary to properly use all fish by-products. Fishery by-products are well known for their content of bioactive compounds, such as unsaturated fatty acids, amino acids, minerals, peptides, enzymes, gelatin, collagen, and chitin. Several studies have reported that fishery by-products could provide significant properties, including antioxidant, antihypertensive, antimicrobial, anti-inflammatory, and antiobesity. Consequently, fish discards are of considerable interest to different industrial sectors, including food, nutraceuticals, medical, and pharmacology. In the food industry, the interest in using fishery by-products is focused on hydrolysates as food additives, collagen and gelatin as protein sources, chitin and chitosan to form edible films to protect food during storage, and oils as a source of Omega-3 and useful as antioxidants. Although different studies reported good results with the use of these by-products, identifying new applications in the food sector, as well as industrial applications, remains necessary.

## 1. Introduction

The aquaculture and seafood processing sectors play an important role in the economic growth of the nation. In 2020, the global capture fisheries production reached 90.3 million tons (an estimated value of $141 billion); 49% of this amount was related to the following seven countries: China, Indonesia, Peru, India, Russian Federation, the USA, and Vietnam. Additionally, 85% of the total capture production represents finfish [1], representing an increase in the demand for nutritious foods that can be beneficial to health since fish is an excellent alternative, particularly in developing countries [2–4]. The fish processing industry generates significant solid wastes in different proportions: muscles (10%), skin and trimmings (3.5%), thorns (9%–15%), heads (10%), guts (12.5%), and scales (3.5%) [2, 5], representing 60% of the total seafood [3]. Due to the problematic effects of climate changes and scarcity of natural resources, improving technologies to process fishery by-products is an interesting topic for many researchers, especially those for the extraction of bioactive compounds [6].

The use of this waste can be a great production opportunity for the fishing and seafood processing industries with great advantages, and the main one is the creation of value-added products, increasing the total utilization of fish and being economically beneficial for industries. Additionally, the recovery of these by-products allows the availability of valuable bioactive compounds present in them and, consequently, major availability for the growing population. Finally, the reduction of environmental hazards is an important consequence of the recovery of fishery by-products [7]. Actually, different fish processing industries are using the fishery wastes as raw materials for fish meal preparation or direct feed material in aquaculture systems, which gives less profit to seafood industries [8]. However, there is still a part which goes to landfill or incineration, resulting in environmental, health, and economic damage [9, 10].

The different fishery by-products (intestine, bones, viscera, etc.) have few possibilities to be commercialized, they are sources of interesting compounds such as amino acids, enzymes, peptides, and collagen, having the possibility to be used for the development of high-value-added products and exploited in different fields [11]. These by-products have great potential for higher-value applications in the nutraceutical, pharmacy, food, cosmetic, and medical sectors; also, they are an excellent alternative source for the biodiesel and biogas industries [12]. The use of dispersant as marine oil is gaining popularity, and the development of more effective and sustainable dispersant is a promising application. The use of viscera and head wastes to produce hydrolysates is one of the most known applications with promising results, and shellfish wastes are used to make calcium lactate and protein hydrolysate to improve animal feed [13]. The demand for fish consumption will soon increase with the number of fishery by-products, which will stimulate the search for new applications to recover discards and subsequent financial and environmental benefits [5].

This review is aimed at summarizing the different fishery by-products generated by the fish processing industry and describing their significant components and benefits, as well as new trends and applications in the food sector. Furthermore, this review is aimed at expanding the significance of the revalorization of fishery by-products, showing real studies related to these by-products, and encouraging continuous research, development, and innovation dependent on the situation of each region.

## 2. Main Fishery By-Products

**Figure 1:**
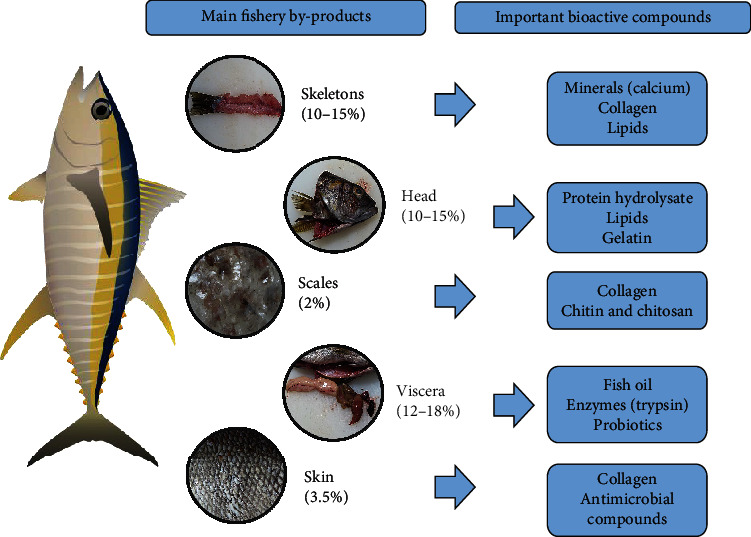
Most common fishery by-products and their most important components.

Fish is a vital source of high-quality protein; it is consumed in either fresh or processed form in different parts of the world. The total marine biomass generates more than 50%–70% of wastes, which are considered secondary raw materials, and comprises different parts, including skin, bones, scale, heads, and viscera [7]. Recovered products possess functional and bioactive properties that are significant for different industries, such as food, agricultural, cosmetic, pharmaceutical, and nutraceuticals ([14]). The most common fishery by-products are presented in Figure 1, and their approximate percentage in whole fish and the valuable compounds present are shown in Table 1. The description of each by-product is presented below.

### 2.1. Skin

Recently, gelatin extraction from fish skin has been a subject of great interest for several researchers [25]. Fish skin is a rich source of collagen and gelatin and the content can reach up to 50% yield, which can be obtained from pure purification, also is a good source of antimicrobial compounds that contribute to the protection against pathogen attacks, such as proteins, lysozyme, immunoglobulin, and lectins [16, 26]. Since it represents a substitute for porcine collagen and skin owing to religious reasons or some diseases (e.g., mad cow disease and foot-and-mouth disease), which limit the use of animal-derived collagen, fish skin is gaining more popularity over time [15].

Fish skin surface is fully covered with scales, which contain protein with 2–20 amino acids, including glycine, glutamic acid, hydroxyproline, arginine, and aspartic acid as the most abundant [27]. However, a problem with this by-product is its heterogeneity in different aspects, including the species of origin, different storage conditions, and, in some cases, the skin may be mixed with the bone or other by-products. Therefore, appropriate management should include proper reusing of these by-products [28].

### 2.2. Bones

Typically, the calcium content in regular diets is deficient; improving the intake of this mineral is necessary to promote the consumption of small fishes or calcium-rich supplements [16]. Fish bones are considered valuable for their mineral and micronutrient contents, primarily for their calcium contents, which can reach until 234 g/kg dry bone, and they are presented in the forms of hydroxyapatite (HA) and calcium carbonate; in their active form, present in fish bones, they can be easily absorbed by the human body [26, 29].

Bones can be used in the synthesis of different products like glue, toothpaste, skin care products, and thermally stable biocomposites [30]. The HA from Tenggiri Fish (*Scomberomerus guttatus*) bones was used as an abrasive material in a toothpaste formula in a concentration of 50%, and the final product had a shelf life of 21 days and maintained an overall acceptance in terms of color, aroma, taste, and texture [31]. Another study prepared calcium supplements from fish by-products with alkaline treatments, measuring calcium bioavailability and nutritional value. The authors reported that fish bone powder is mainly constituted of nine essential amino acids and fatty acids like oleic acid, palmitic acid, and gondoic acid [32]. These studies demonstrate the significance of fish bone powder and how its content can be exploited in the food industry for the development of new innovative food products. [33].

### 2.3. Scales

Fish scales represent a significant number of discards from fish industries, since they are removed before filleting. Their surface layers contain HA, an inmost layer of Type I collagen, and low concentrations of minerals such as calcium, magnesium, phosphorus, sodium, and sulfur [16]. They represent an average of 2% of fish body weight; however, fish scales are not biodegradable, and their management could be difficult.

Fish scales are also used in water treatment, and Irawan et al. [34] obtained chitosan from Papuyu (*Anabas testudineus*) scales and evaluated their efficiency in removing iron as a natural coagulant for groundwater treatment. The authors reported that the use of chitosan aided in decreasing the iron concentration from 11.80 to 3.43 mg/L (around 71% removal); moreover, they observed that the coagulation treatment with chitosan was more efficient than using commercial coagulants, representing an interesting option to consider the revalorization of fishery by-products.

### 2.4. Heads

Fish heads are frequently discarded without knowing their high protein content. They can be used to produce products, including fishmeal, fish protein hydrolysates (FPHs), fish paste, feed supplement, or fertilizers. Moreover, fish heads are a source of Type I collagen, which can be an alternative for the lack of collagen in terrestrial vertebrates [26, 35].

Studies employed heads from different fish species and reported them as valuable source for obtaining FPHs; such as seabream (*Sparus aurata*), sea bass (*Dicentrarchus labrax*) [36], skipjack tuna (*Katsuwonus pelamis*) [37, 38], bigeye snapper (*Priacanthus tayenus*) [39], and *Chlorurus sordidus* [22]. He et al. [40] obtained FPHs from yellowtail kingfish heads (previously minced and mixed with water to form a mince-water slurry), using different treatments. Firstly, a conventional chemical alkaline treatment using a 2.5 M NaOH solution adjusting the mince-water slurries to pH 14. An enzymatic process was performed using commercial protease, Flavourzyme 500 MG in mince-water slurries and, finally, a microwave-intensified process for both treatments was performed at 100°C, irradiation power of 500 W for 20 min at atmospheric pressure. Microwave intensification increased the production yields of the enzymatic process from 42% to 63%, and the chemical process from 87% to 98%, showing that with an appropriate extraction method, fish heads can be a good source to produce FPHs. For its part, Li et al. [41] showed that rainbow trout heads are a good source for isolating protein owing to their high protein content (29%) and unsaturated fatty acids, including polyunsaturated fatty acids (PUFAs) (38.6%) and monounsaturated fatty acids (MUFAs) (38.6%), which is highly similar to results observed for Atlantic salmon heads.

### 2.5. Viscera

Fish internal organs, called viscera, represent approximately 20% of total body mass and consist of organs such as the stomach, liver, intestines, pyloric caeca, pancreas, and hepatopancreas. They are considered as important protein source and are used to develop new value-added products. Additionally, they contain vital bioactive materials that enhance the value of fish by-products and represent a rich source of different digestive enzymes [15]. Proteases and peptidases, carbohydrases, lipases, and nucleases are the main enzymes in fish viscera that can be used in different industrial applications.

Moreover, fish oil can be extracted from viscera using different processes, such as rendering, pressing, microwave-assisted extraction, supercritical fluid extraction, solvent extraction, autolysis, and enzymatic hydrolysis. Among them, supercritical carbon dioxide (CO_2_) is the most promising green extraction method, whereas other methods are still being developed and need more studies or new applications [14, 35, 42]. Although fish enzymes are not directly applied in functional foods and nutraceuticals, they can be used at a large scale in bioactive component production [15].

### 2.6. Swim Bladder

Swim bladder is an internal organ that aids fishes to control their movement in the water, and their primary role is to control buoyancy in order that fishes do not expend energy to maintain their vertical position in the water column; other roles associated with this organ are respiration, hearing, and sound production [43]. Swim bladder contain high collagen protein, playing a significant role in the process of healing for new tissue growth, it has been applied in the biomedical field as tissue engineering scaffolds [44–47].

Additionally, some studies have reported collagen extraction from swim bladders of different fish species, including yellow drum fish (*Protonibea diacanthus*) [48], sea bass [46], pangasius [49], crucian carp [50], and Gulf corvina [51]. Other studies have started to identify new benefits from swim bladders; Howaili et al. [45] prepared a scaffold with decellularized fish swim bladder and colloidal silver nanoparticles to evaluate its antimicrobial activity; the scaffold showed appropriate antimicrobial activity and a good barrier. Likewise, Li et al. [24] reported that swim bladder collagen from grass carp has proteins with superior foaming and emulsifying properties compared with mammalian-based collagen, such as chicken. The authors concluded that the swim bladder is a promising source of collagen that could be successfully applied in food, cosmetic, and medical fields; further research is needed to identify other bioactive compounds present in swim bladder and apply in products of the different fields.

## 3. Composition of Fishery By-Products

The composition of each by-product can be widely variable. It depends on different factors, including age, species, nutritional value, and health; however, in general, fishery by-products have a proximal composition quite similar to products for direct consumption [18]. The proximal composition of by-products from different fish species is detailed in Table 2.

Fishery by-products have significant water content, ranging from 50% to 80%. Their protein content ranges from 15% to 30% (depending on the fish species) and contains essential amino acids, such as glycine, lysine, valine (Val), and phenylalanine (Phe), which can improve the nutritional quality of products in food and nutraceutical industries [16]. The fat content of fish by-products is approximately 0%–25%, wherein the most important and studied components are Omega-3 fatty acids due to its beneficial effects against cardiovascular diseases and enhance the immune system [16, 33]. These fatty acids cannot be synthesized by the human body; therefore, identifying supplements enriched with Omega-3 fatty acids is necessary [18]. Regarding the mineral content, 60%–70% of the total minerals present in fish are in bones or skeletons, being calcium, phosphorus, and HA being the most abundant [60]. However, to use it for a different application, calcium has to be converted into an edible form by thermal treatment with acid solutions or by superheated steam cooking [16].

## 4. Nutrients and Bioactive Compounds in Fishery By-Products

Fishery by-products represent a wide variety of possibilities and challenges that are essential to study, and the isolation of different bioactive materials, including calcium from fish bone, crude enzymes from viscera, and proteins from skin and head allowing industrial processes can be more effective and sustainable [2, 15].

### 4.1. Bioactive Peptides

Several bioactive peptides are noted in fish proteins, these peptides have been studied and showed to have vital properties, including antihypertensive, antithrombotic, immune modulatory, and antioxidant properties, being an interesting alternative to be used as functional ingredients for different applications (bakery products, soups, and infant food formulas). Its antioxidant activity can inhibit lipid peroxidation and remove reactive oxygen species [16, 61, 62].

However, these peptides are inactive in the structure of native protein, and they must be liberated by proteolysis (*in vivo* digestion) or hydrolysis (*in vitro* by enzymes) to promote their biological activity. Enzymatic hydrolysis is preferred in food and pharmaceutical industries since it does not result in residual organic solvents or toxic chemicals, which could affect their nutritional content [18]. Nevertheless, it is said that these bioactive peptides could have certain toxicity levels within them, and Lutfi et al. [63] evaluated the toxicity level of *Biophytum umbraculum* ethanolic extract for its potential utilization in aquaculture; the analysis showed the presence of alkaloids, steroids, triterpenoids, saponins, flavonoids, total phenols, and tannins. The authors concluded that the extract could be used for the reproductive, nutritional, and health aspects of fish. In general, it is important to be careful in the functional application of these peptides, even though the toxicological data from them is still very few and the analytical methods to guarantee the products' safety are still ongoing.

These peptides exhibit little or no toxicity; there are still some studies ongoing to prove fish peptides' safety and their application in foods. Nesse et al. [64] assessed the safety of supplementation with Amizate® (proprietary protein hydrolysate preparation from Atlantic Salmon (*Salmo Salar*)) in malnourished children between 6 and 8 years old. The administration of the supplement did not present adverse effects, demonstrating that the consumption of Amizate® is safe to be included in children's diets. Since fishery by-product is a worthy subject to study, clinical essays with similarities to the human organism are becoming increasingly necessary; therefore, the use of mice for *in vivo* assays will be more frequent. The effects of FPHs in human health are discussed below.

#### 4.1.1. Antioxidant Activity

Oxidation is a chemical process that normally occurs in the human body, where a compound loses electrons and is oxidized, and another gets them and reduces. However, when the oxidation process becomes more intense or prolonged, oxidative stress associated with different deteriorative processes, including protein damage, lipid peroxidation, enzyme inactivation, and DNA breakage, may occur. These processes favor diseases or pathologies, including cancer, heart disease, rheumatoid arthritis, and aging [65].

Antioxidants are the most employed molecules against free radicals for their ability to neutralize them and stop their formation [66]. They are typically composed of peptides of 3–16 amino acids, of which histidine or tyrosine and methionine, cysteine, tryptophan, and lysine are the main amino acids with antioxidant activity in their free form [10]. The most described mechanism of antioxidant peptides includes inhibiting lipid peroxidation, scavenging of free radicals, and metal chelating activity; however, no exact mechanism has been described to date [66]. Peptides with higher antioxidant activity have been isolated from different fish by-products with possible uses in food, pharmaceutical, nutraceuticals, and even medical fields, indicating an alternative for synthetic antioxidants [67].

Recently, studies have shown how the production of protein hydrolysates can be a valuable source against different oxidation processes. Salmon protein hydrolysate is a potential source of antioxidant peptides when released in the gastrointestinal tract; however, their amino acid sequence and quantification can vary internally [68]. The antiallergic activity of purified peptides from Atlantic salmon (*S. salar*) viscera was recently studied by Wang et al. [69]; they reported peptide fractions with antiallergic activity up to 89.40% representing a novel functional ingredient for developing special food for individuals with allergies. Additionally, the antioxidant activity of FPHs has been evaluated in the medical field, employing different cloned human cells related to diseases such as colon adenocarcinoma [70], human colorectal carcinoma [71], and human umbilical vein endothelial cells [72]. This research with others which evaluated the antioxidant activity of different FPHs is summarized in Table 3.

#### 4.1.2. Antimicrobial Activity

Different antimicrobial peptides can be found in fish, such as defensins, cathelicidins, hepcidins, and peptides derived from histones and piscidins. They work as a general protective in fish skin, capable of targeting different pathogens since they have low specificity and do not develop any resistance towards them [77]. Peptides mainly consist of a 12–45 amino acid chain, positively charged for the presence of NH_3_^+^ groups (the more NH_3_^+^ groups the peptide has, the greater its antimicrobial activity). When the NH_3_^+^ groups present in the peptides interact with the surface of a microorganism (negatively charged), a change of charges occurs, resulting in leakage of cellular substance and subsequent in the destruction of the bacteria, the greater the number of positively charged amino groups in the structure of a biopolymer, the greater its antimicrobial activity [77, 78].

Most fish peptides possess antibacterial properties against different Gram-negative bacteria, including *Aeromonas hydrophila*, *Klebsiella pneumoniae*, *Salmonella enterica*, and *Salmonella typhi*, and Gram-positive bacteria, including *Streptococcus iniae*, *Micrococcus luteus*, *Staphylococcus aureus* (*S. aureus*), and *Bacillus cereus* [18]. Antimicrobial peptides are of considerable interest for new antibiotic development in the pharmaceutical field and antimicrobial agents for the food industry. They are potential candidates for the pharmaceutical field since they can be used as antibacterial, antiviral, antifungal, immunomodulatory, and antitumor agents; this is possible owing to their selectivity to the membrane of the pathogen because of the differences in the interactions of existing charges caused by different lipids [65].

Trang and Pasuwan [79] reported the potential antimicrobial activity of protein hydrolysates of Nile tilapia by-products extracted via enzymatic hydrolysis and at specific concentrations of approximately 3%–8% mg extract powder/mL. Pezeshk et al. [80] detected antibacterial activity in proteins hydrolyzed from yellowfin tuna (*Thunnus albacores*) viscera, wherein low-molecular-weight peptides possessed better antimicrobial activity than high-molecular-weight peptides at a minimum inhibitory concentration (MIC) of 0.5 mg/mL.

Since studies have reported that FPHs may be a significant source of antioxidants and antimicrobial compounds, medical fields have been recently interested in the possible use of these bioactive compounds as a promising alternative in the prevention and treatment against certain diseases. In the case of their antimicrobial activity, FPHs play a role in the gut microbiota by increasing the content of beneficial bacteria and decreasing those related to inflammatory diseases, allergy, and even obesity [81]. However, only few studies of fish by-product hydrolysates were applied to the gut microbiota, with all studies agreeing that peptides can be valuable as antimicrobial agents. Some recent studies on the effects of FPHs on the gut microbiota are summarized in Table 4.

#### 4.1.3. Antihypertensive Activity

Hypertension is a chronic disease that affects millions of individuals worldwide, which can be a risk factor for cardiovascular diseases, and its anomalies such as coronary artery disease, stroke, and cardiac arrhythmia can affect kidney functions [7]. Angiotensin 1-converting enzyme (ACE), an enzyme associated with hypertension, is a dipeptidyl carboxypeptidase responsible for the conversion of Angiotensin I to II. This enzyme has a physiological effect on narrowing blood vessels and increasing blood pressure [10]. Therefore, it is necessary to inhibit ACE to regulate blood pressure and treat hypertension; synthetic inhibitors, including lisinopril, captopril, losartan, atenolol, and hydralazine, are used for medical treatments; however, they may have adverse effects, including inflammatory response, dry cough, taste disturbance, skin eruptions, or angioneurotic edema [85].

Peptides from fishery by-products have been reported as inhibitors of ACE activity and are related to the degree of hydrolysis, which depends on different factors, including protein substrate, proteolytic enzymes, and their concentration. This is possible for the nitrogen or carbon terminals of peptides that change the ACE activity [77, 86]. Vázquez et al. [87] optimized the production of protein hydrolysates from *Scyliorhinus canicula* wastes and obtained peptides with potential antihypertensive and antioxidant activities, the optimal conditions for proteolysis were at 60.8°C/pH of 8.9 and 64.6°C/pH of 9.4 for Esperase and Alcalase, respectively. Moreover, Pérez-Escalante et al. [88] established antioxidant and antihypertensive properties of rainbow trout muscle protein hydrolysates, and the best results were obtained after 1 hour of hydrolysis which were a scavenging and ferric-reducing power of 2.65 ± 0.07 *μ*M Trolox equivalents and 32.12 mM Fe^2+^ equivalents, respectively. This indicated a promising alternative in the research concerning bioactive peptides for cardiovascular treatment. A summary of more studies reporting antihypertensive activity of FPHs is presented in Table 5.

#### 4.1.4. Anti-Inflammatory Activity

Inflammation is a mechanism that promotes healing; however, its excess in a specific site can lead to tissue damage or implant rejection. When inflammation occurs, macrophage cells release nitric oxide and other inflammatory mediators (e.g., prostaglandin E2 and cytokines) to avoid complications and heal the injury. However, regulating the macrophage behavior and the inflammatory process is significant because their excess could contribute to several life-threatening diseases [93].

Fish by-products have been reported to present anti-inflammatory properties and could be included in functional foods or within nutraceutical formulations, representing an alternative to synthetic drugs for the prevention or treatments of inflammatory diseases [94]. Fish peptides from by-products have lower molecular weight and short chain sequence (2–20 amino acids), with hydrophobic amino acids being possible radical scavenging mechanisms [7, 66]. Typically, anti-inflammatory peptides have positively charged amino acids in the N- and/or C-terminal positions, of which the most common are arginine, lysine, and histidine; however, there are some anti-inflammatory peptides with negatively charged amino acids, including glutamine and arginine. Peptides with hydrophobic amino acids (alanine (Ala), Phe, leucine (Leu), Val, and isoleucine (Ile)) can reduce nitric oxide production, which is an inflammatory mediator, and this is explained by their attraction and reactivity with the cell membrane, which promotes anti-inflammatory effects. Nevertheless, this mechanism has been suggested to be used not only as an indicator but also as an anti-inflammatory activity in medical and pharmaceutical fields [95]. Regardless of the amino acid charge, bioactive peptides from fishery by-products show an interesting anti-inflammatory potential [7, 96]. More studies on anti-inflammatory activity are presented in Table 6.

#### 4.1.5. Antiobesity Activity

Obesity is a disease that is considered as a disorder of lipid metabolism and is associated with other chronic diseases, including hyperlipidemia, hypertension, coronary heart disease, and Type 2 diabetes. In the digestion of triglycerides, some enzymes (such as pancreatic lipase) can hydrolyze dietary triglycerides into monoglycerides, free fatty acids, and other small molecules that are easily absorbed and resynthesized into new triglycerides, leading to obesity. Therefore, the inhibition of lipase is significant to prevent obesity [102, 103]. However, the antiobesity activity of fish peptides can be explained by the sequence of its amino acids, particularly if essential amino acids are present since they can reduce the expression levels of transcription factors of obesity, such as peroxisome proliferator-activated receptor *γ*, sterol regulatory element binding protein-1, and CCATT/enhancer-binding protein *α* [95].

Since this property has been recently discovered, only few studies of these subjects are available. However, the majority of them agree that FPHs have a potential activity against obesity and related diseases, particularly Type 2 diabetes, owing to its glycemic control. Mizushige et al. [104] studied the effects of Alaska Pollack (*Theragra chalcogramma*) hydrolysate digested artificially with pepsin and pancreatic on white adipose tissue and skeletal muscle of rats. The rats that were fed with FPH showed lower weight of white adipose tissue and higher weight of soleus muscle than the control group, this could imply a possible antiobesity activity by the reduction of appetite. Moreover, Woo et al. [105] explained that fish peptides regulate hepatic lipid metabolism factors and enzymes; the authors fed mice with collagen fish peptide-based diet and observed decreased lipid levels and reduction in body weight and visceral adipose tissue weight. The study reported that the antiobesity effects of fish peptides are highly related to the molecular weight or structure of the amino acids, which depends on the type, source, and preparation of the peptides. A resume of more recent studies of FPHs and their effects on obesity is presented in Table 7.

### 4.2. Lipids

Oils and fats represent a considerable fraction of finfish processing waste, approximately ranging from 2% to 30% in the form of fish oil. The amount depends on different factors, including species, fat distribution in fish parts, age, sex, nutritional status, and health [12, 18]. To recover significant amounts of fish oils, discards such as heads and viscera (particularly the liver) are the most employed [60]. A resume of fatty acid profiles of by-products of different fish species is shown in Table 8.

Marine oils are rich in PUFAs, of which the most recognized are the n-3 PUFA family of alpha-linolenic acid (C18:3n-3) and elongated and desaturated products, including eicosapentaenoic acid (EPA, 20:5n.3), docosapentaenoic acid (22:5n-3), and docosahexaenoic acid (DHA, 22:6n-3). Also, marine oils are a good source of vitamins A, D, and E, and their consumption can help to maintain a good help and in the prevention of different diseases, including high blood pressure, coronary heart diseases, cancer, dementia, and depression [113–115]. The antimicrobial activity of these peptides and fish oil extracted from *S. salar* wastes were evaluated against Gram-positive and Gram-negative pathogens. Results showed an inhibition effect with MICs of 25% and 12.5%, respectively. This indicated that fish oil extracted from the waste remained active against both bacterial strains owing to cellular membrane alteration [116].

However, the high PUFA content makes fish highly susceptible to lipid oxidation, thereby affecting its quality and nutritional value, such as the decrease in caloric content or the loss of essential fatty acids and lipid-soluble vitamins. Lipid oxidation is related to damage of cell structures, which leads to their death, and this can have consequences in aging, mutagenesis, and carcinogenesis [117]. To extract these compounds and apply them to food matrices, it is significant to select an ideal extraction method that does not affect the quality and stability of fish oils. Wet pressing method, supercritical fluid extraction, and enzymatic extraction are the most common extraction methods [60]. A recent study by Aitta et al. [118] employed enzymes Alcalase®, Neutrase®, and Protamex® to recover fish oil from different Baltic herring (*Clupea harengus membras*) by-products such as heads, fins, tails, and viscera; the major oil yield was in a range of 4.6 to 6.1 g/100 g by-products. The extraction with Protamex® resulted in oils with lower volatile contents, but more studies are still needed to optimize the enzyme-assisted oil extraction to improve yields and quality of oils.

### 4.3. Minerals

The content of minerals, such as calcium, phosphorus, and HA, in fish bones is approximately 60%–70%. Calcium deficiency in the diet is a common problem, and the consumption of whole fish can be a valuable option to consider [16]. Minerals can be divided into the following two groups: major minerals (calcium, sodium, and phosphorus) and minor minerals (iron, zinc, copper, manganese, and selenium) [61].

About major minerals, calcium is the major mineral present in fishery by-products, the HA is a calcium phosphate used for biomedical application and can be found in fish skeletons. Abdullah et al. [29] obtained HA from tilapia fish bones and scales by the calcination method at three temperatures: 1000°C, 900°C, and 800°C, where the highest temperature resulted in a dense structure and the removal of all impurities, making 1000°C the best calcination temperature to obtain HA powder with high ash, fat, and protein content. Additionally, the bioavailability of Ca from aquatic sources is a better alternative than milk. Phadke et al. [7] indicated that peptides of oligo nature extracted from bones of hoki fish showed calcium-binding properties that could inhibit calcium salt formation.

Regarding microminerals, they are essential for the growth and maintenance of a normal health of fish, and a deficiency could cause a decrease or loss of enzymatic activities [119]. Iron and zinc are the main minor minerals present in fishery by-products, particularly in the liver, gills, and guts [109]. Iron is involved in different biochemical processes, such as oxygen transport, energy production, and cell proliferation; however, a large part of the world's population is iron deficient. Therefore, some options to incorporate iron to diets can be salts, metal-chelating agents, and iron-chelating peptides [61]. Zinc is the second most abundant micromineral after iron and is involved in several biological functions of more than 300 enzymes such as RNX nucleotide transferases, alkaline phosphatase, and carbonic anhydrases [119].

Other essential microminerals present in fishery by-products are copper, manganese, and selenium, and the levels of each mineral can be approximately 600 *μ*g/100 g, wherein the liver and gills presented the highest amounts [109]. Jaziri et al. [120] found different minerals (zinc, selenium, iron, copper, manganese, chromium, cadmium, and lead) in purple-spotted bigeye (*Priacanthus tayenus*; Ricardson,1846) and barracuda (*Sphyraena obtusata*; Cuvier, 1829) by-products, where Zn was the most abundant in skin, bones, and fin for both species (1386 to 2408 *μ*g/100 g in purple-spotted big eye and 1502 to 6039 *μ*g/100 g in barracuda). However, high levels of these minerals could affect the safety of these by-products for their recovery, and Aljabryn [121] found high levels of arsenic (As) in seven major commercial marine fishery species in a local market in Saudi Arabia, ranging from 0.16 to 4.30 mg/kg; whereas the other heavy metals were within the standard limits.

## 5. Applications in the Food Field

With global population growth, fishery industries have been increasing their production and subsequently the amount of their by-products. The valorization of fish discards is a great challenge for the industry and requires novel technological approaches. However, there are some restrictions that can be challenging, such as off-odor, low functionality, high costs, and low consumer acceptance [10].

**Figure 2:**
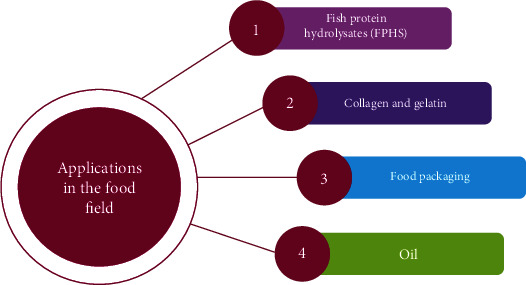
Application of fish by-products in the food field.

In the food and nutraceutical industries, fishery discards represent an interesting source of high-added value compounds, including HA, collagen, gelatin, lipids, enzymes, hydrolysates, and bioactive peptides [16]. Different technologies have been used to obtain new food products from these discards. Huang et al. [122] developed a novel extrusion-hydroextraction process for the extraction of collagen from the tilapia scale. The extruded product had 2–3 times higher protein extraction yields compared to nonextruded samples, with possible applications in the food, medical, and cosmetic industries. He et al. [123] employed high-intensity pulsed electric fields (PEF) and could extract chondroitin sulfate (CS) from fish bones which are known for their activity of anticoagulant, antitumor, and prevent artery hardening and arthritis. Ferdosh et al. [124] extracted fish oil from *Thunnus tonggol* heads using supercritical CO_2_, recovering PUFA, such as DHA, Omega-3, and Omega-6, in each fraction obtained. Other traditional technologies have been combined with another emerging technology to increase their efficiency. Richa et al. [125] designed and developed a hybrid system with an ohmic heater and solar energy for drying fish muscles, and the outcome was a final product with less moisture content maintaining the nutritional quality of the fish. He et al. [126] combined PEF with semibionic extraction to increase the extraction of calcium, collagen, and CS from fish bones, reporting that the combination of both technologies made a great extraction compared with other traditional methods. Saidi et al. [127] employed a combined process associating ultrafiltration and nanofiltration membranes to increase the production of tuna by-product protein hydrolysate. The obtained fractions can be used as a source of high nutritional quality, in human nutrition and possible application in aquaculture diets. Recent studies that used different fishery by-products to isolate significant bioactive compounds and apply them to some food systems are in Figure 2 and summarized in Table 9.

### 5.1. FPHs

Protein hydrolysis is a widespread method used to extract protein from meat and fish residuals, consisting of breaking down proteins into smaller and more water-soluble peptides and free amino acids [2]. Owing to their significant properties, including water holding, oil absorption, and foaming capacity, as well as their protein solubility and gelling and emulsification ability, FPHs show potential applications as functional ingredients for pharmaceutical and clinical applications [16].

The consumption of protein hydrolysates and peptides from fish by-products can improve human health and help prevent chronic diseases, which is a current global concern [65]. At present, FPHs have been applied in different food systems such as cereals, cookies, desserts, and meat products with promising results; however, there is still few information about how to scale up the obtained results of FPH food applications [139]. For the obtention of FPHs, chemical and biological methods are the most used in industrial fields. A resume of some studies in fishery by-products is presented in Table 10.

#### 5.1.1. Chemical Hydrolysis

The conventional chemical hydrolysis is based on sample treatment in acid or alkaline solutions accompanied by high temperatures or, in some cases, with high pressures until reaching the desired degree of hydrolysis [139]. This method removes oil and water from the substrate, concentrating the intact protein. Some advantages of this method include its fast processing and low cost. However, recently, chemical hydrolysis seems to affect the quality of FPHs, the formation of undesirable metabolites and nitrogenous compounds, the functionality of biocompounds, the loss of freshness, and the destruction of essential amino acids. This is unfavorable for the food and drug fields; therefore, this method is currently used for low-value products, including fertilizers, or for the growth of lactic acid bacteria [144].

The acid hydrolysis is the more conventional, it uses extremely acidic solutions at high temperatures and, in some cases, at high pressures for a prolonged time. Conversely, alkaline hydrolysis needs a pretreatment, which consists of protein solubilization by heat followed by the addition of alkaline agents (e.g., calcium, sodium, or potassium hydroxide), adjustment of the desired temperature, and hydrolysis for several hours [2]. Over time, the use of chemical hydrolysis has been reduced since acid and alkaline treatments are difficult to control and generate large amounts of residues that affect the quality and functionality of isolated proteins along with the destruction of essential amino acids during the process [10].

#### 5.1.2. Enzymatic Hydrolysis

Enzymatic hydrolysis has been a subject of interest for different industries owing to its advantages over chemical hydrolysis. Unlike chemical hydrolysis, this method is easy to control, and the results are more precise and create no residual reactions or decrease in the quality and functionality of the obtained peptides. Consequently, food industries are employing the use of FPH-producing enzymes for the production of new functional products [139]. Food industries use enzymes from different sources: proteases such as Alcalase®, Flavourzyme®, and Protamex®, which are derived from various microorganisms; enzymes from plants such as papain, bromelain, and ficin; and enzymes from animal sources such as pepsin, chymotrypsin, and trypsin [145].

The enzymatic process can be performed using the endogenous or exogenous enzymes: endogenous enzymes perform the natural process in the digestive tract; however, this process takes too much time because it is influenced by different factors, including age, season, sex, and species. Therefore, the use of exogenous enzymes is the best choice to produce FPHs since it is more specific and is possible to obtain a better control in the hydrolysis process [2, 10]. Although the results of enzymatic hydrolysis are successful in the laboratory, they are not the same in the industrial level owing to their high cost and are not economically viable [146].

### 5.2. Collagen and Gelatin

Collagen is the most abundant protein present in the extracellular matrix of connective tissues in the body. Collagen production decreases with age and a bad diet; consequently, it is necessary to supplement the human diet with food and medical products with high collagen levels [61]. Bovines and porcines are the major sources of collagen, accounting for almost 70% of the total collagen worldwide. This has been a matter of concern in recent years owing to the constraints of some religions, mainly Islam and Judaism, wherein the consumption of these animals is prohibited. Therefore, marine collagen is becoming highly attractive as a natural source [147, 148]. Fish skin and bones are excellent sources of collagen and gelatin, and their properties are of considerable significance for several application fields [18, 149].

#### 5.2.1. Collagen

Collagen is a fibrous protein, which is the major structural protein in the connective tissue of animal skin and bone. It is a glycoprotein that contains small amounts of galactose and glucose and is found in abundance in mammals in different fish parts, such as the skin, tendons, cartilages, bones, and connective tissues [9]. At least 28 types of collagens exist depending on the domain structure and their superstructural organization; however, major groups include Types I, II, and III, accounting for 80%–90% of the total collagen types. Type I is the most abundant and can be found in bones, skin, tendons, and organs. Type II can be found in cartilages, and Type III is observed in reticular fibers, blood, and skin [150]. The three most abundant types of collagens (I, II, and III) and their sources of origin in fishery by-products are shown in Table 11.

Compared with mammalian collagen, marine collagen presents a comparable or slightly lower molecular weight and a lower denaturation (melting) temperature of approximately 20°C–35°C for most fish species with higher collagen values [9, 18]. To estimate how much collagen is present in fish, hydroxyproline is used as an indicator since this amino acid is a particular element of the protein collagen. Hydroxyproline plays a significant role on the thermal stability of fish collagen and has a lower molecular weight; however, its content is much lower than that of mammalian-based collagen [153, 154].

In the global protein market, collagen is highly promising owing to its future applications in food and beverage industries; the focus is on the preparation of functional foods and beverages as well as food supplements [10, 155]. Its high water absorption capacity makes collagen an ideal supplement for texturizing, thickening, and gel formation. However, marine collagen remains underexploited, and its applications are lower than those of mammalian collagen [9]. Therefore, the proper use of fishery by-products and the recovery of their components are significant points to consider for future research.

#### 5.2.2. Gelatin

Gelatin is the denatured derivative of collagen. It consists of fractions of proteins and polypeptides of different molecular weights. The quality of gelatin depends on various factors, including the species of origin and the extraction procedure, wherein the most common processes can be chemical processes (acid or alkaline treatment) or biotechnological by the use of enzymes [17, 156].

Lv et al. [157] reported that gelatin presents abundant amino acids, including glycine, proline, Ala, and hydroxyproline. These amino acids form a branched chain, which is responsible for the anabolic processes in protein metabolism and muscle growth following exercise [158]. Some authors affirmed that compared with mammalian gelatin, the amino acid content present in fish gelatin is excessively reduced, and its application as a gelling agent is limited. However, it can be solved by adding some polysaccharides to form polyelectrolyte complexes [153].

Despite its limited application, gelatin can be used for improving different foods and beverages. In the meat industry, it can be used as an emulsifier or fat binder. Furthermore, it is used in food creams (e.g., ice cream, jellies, and cream pies) as a stabilizer, and it is used in foods formulated with high protein levels that are beneficial for patients with diabetes [152]. Additionally, owing to its film-forming properties, gelatin is used to produce biodegradable plastic films as food recovering for a prolonged shelf life [35]. Gelatin has the ability to reversibly change from sol to gel and swell even when placed in cold water, a property that other thickeners like xanthan gum, carrageenan, or pectin do not have [148].

Furthermore, in the nutraceutical and pharmaceutical fields, gelatin is used in the elaboration of products, including capsules, tablet coatings, and emulsions, and the microencapsulation of compounds, including vitamins and drugs [152]. In the biomedical field, collagen, and gelatin are used as drug carriers and in tissue engineering, cartilage regeneration, and wound healing [15]. Since gelatin possesses essential amino acids from fish collagen, thereby promoting cell adhesion and differentiation, natural gelatin scaffolds are safer, more compatible, and with less rate of immune reactions than synthetic scaffolds [18].

### 5.3. Food Packaging

The application of fishery by-products in the packaging sector is a relatively new issue, which has arisen owing to the need to implement more sustainable practices in the fishery sector, involving the valorization of by-products and discards [9]. Fish by-products have polymers that are favorable substitutes of synthetic polymers to produce bioplastics; however, some are not as biodegradable as they claim to be. To design a sustainable food packaging, it is necessary to use polymers that present the required properties, such as biodegradability, biocompatibility, low toxicity, and renewability [159].

#### 5.3.1. Chitin and Chitosan

Chitin and its derivatives (e.g., chitosan and chitooligosaccharides-CO-S) are marine polysaccharides that possess biological and physicochemical characteristics and provide them with properties that are used in several industrial applications, including foods, textiles, cosmetics, and pharmaceuticals [17, 160]. In the food industry, they are widely used to prepare edible films for food packaging that are clear and flexible and provide good oxygen barrier properties. Their mechanism of action consists of the interaction between the positively charged chitin and chitosan molecules and the negatively charged microbial cell membranes. The interaction changes the properties of membrane wall permeability, thereby causing an osmotic imbalance and inhibiting microbial growth [115].

Chitin is one of the most abundant polysaccharides in nature and is the second most abundant high-molecular-weight natural biopolymer after cellulose. The presence of nitrogen in its chemical structure distinguishes it from other sugars. The most common and known sources are shellfish wastes, including shrimps, crabs, and lobsters; cell walls of some fungi (*Zygomycetes*); or they can be obtained using chemical treatments, such as alkaline hydrolysis of chitin from natural sources (e.g., crustaceans or arthropods) [115, 161, 162]. Chitin biomolecules and derivatives have excellent biodegradability and have numerous biological properties, including antimicrobial, antitumor, anticoagulant, antioxidant, antimutagenic, and cholesterol-lowering properties [114].

Chitosan has certain adhesive, antibacterial, antifungal, antiviral, and nonallergenic properties recognized in the field of food preservation and packaging that can be used to avoid chemical preservatives and produce edible films, is nontoxic, and can be biodegraded by lysozyme contained in human body fluids [159]. To evaluate the antibacterial activity of chitosan, Molina-Ramírez et al. [20] extracted chitosan from fish scales of *Prochilodus magdalenae* and prepared a 4.5 wt.% chitosan solution in 2 v.% acetic acid. The solution was added to a banana starch-based film and evaluated in *Escherichia coli* (*E. coli*) and *S. aureus* cultures; positive results were obtained in the *E. coli* culture, particularly owing to the low-molecular-weight characteristics of chitosan. Chitosan has been applied in the field of food packaging too in order to extend the shelf life of food, and Mehebub et al. [163] applied chitosan from shrimp shells by spraying it onto litchi fruits, application of 500 *μ*g/L enhanced phenolic compounds and antioxidant activity in the fruits, representing an alternative against harsh synthetic chemicals for the safe and sustainable litchi production.

### 5.4. Oil

Various studies are aimed at fortifying food products with Omega-3 and thereby increasing the consumption of these lipids. Ghorbanzade et al. [138] added fish oil to a lecithin–oil mixture, deionized water, and glycerol to be added to different yogurt formulations, with a volume of 15 mL emulsion in 100 g yogurt. The authors reported that fish oil did not affect the sensorial properties of yogurt in terms of color, taste, and texture on commercial samples. Moreover, after 21 days of storage, the fatty acids of yogurt showed a significant retention of DHA and EPA of 57% and 12%, respectively; however, in samples without fish oil, the value of this fatty acid was 27% and 6%, respectively. Ainsa et al. [135] used fish oil from sea bass (*Dicentrarchus labrax*) trimmings and added to fresh pasta with the aim of increasing protein and unsaturated fatty acid contents. Compared with commercial pasta, the products showed an effective protection against oxidation for 90 days shelf life, their protein content increased from 12.50% to 19.55%, and their texture properties improved in a softer product and less cooking time.

Moreover, studies have demonstrated the potential of fish oil as an antioxidant. Sellami et al. [164] extracted oil from the waste liver of three ray species and evaluated the extract composition and scavenging activity; the fatty acid profiles exhibited a dominance of unsaturated fatty acids, and the major n-3 PUFAs were EPA and DHA, with contents varying from 3.36% to 5.51% and 9.07% to 30.50%, respectively. Furthermore, the oil contained carotenoids and phenolic compounds displaying antioxidant activity comparable to that of olive oil. Pérez-Palacios et al. [137] added microencapsulated cod liver oil to chicken nuggets and evaluated their effects on the oxidation process. The oil did not affect the sensory attributes of the nuggets; however, it provided protection against lipid and protein oxidation measured using the thiobarbituric acid reactive substance (TBARS) test. The authors affirmed that the inclusion of cod liver oil increased the PUFA content and could be commercially well accepted as a healthy product.

To produce high-quality fish oil rich in Omega-3, a suitable extraction procedure is required, and the most common is wet reduction, which involves the following three steps: cooking at high temperature (85°C–95°C), pressing, and centrifuging. However, recently, different industries have used other methods, including enzymatic reactions with proteases and supercritical fluid extraction. The second process uses moderate temperatures and provides an oxygen-free medium to reduce Omega-3 oxidation during the extraction process [165].

## 6. Conclusion

The demand for fish production will increase over time and subsequently the number of fishery discards. However, if these by-products are not recovered or processed, a loss of vital nutrients, negative environmental effects, and a significant loss in income could occur. These by-products are good sources of bioactive compounds, particularly proteins and lipids, with benefits to human health for different age groups. Therefore, it is highly probable that, in the next few years, different industries will develop new products that are attractive for their benefits and low costs. Different studies have shown the results of these by-product applications in food systems to fortify them with lipids, proteins, or minerals, and thus, foods could present significant properties, including antioxidant, antihypertensive, and antimicrobial properties. Additionally, chitin and chitosan from fish scales and skin are good alternatives to be employed in food packaging and thereby prolong shelf life and protect against oxidative or microbial damage. More studies on compounds from fish by-products are needed to better understand their behavior and possible application in different food products or other applications related to the food industry and, consequently, create new green technologies that would be necessary to recover these discards without any environmental consequences.

## Figures and Tables

**Table 1:** Association between fishery by-products, percentage, and valuable sources.

**By-product**	**Representative percentage (%)**	**Valuable source**	**References**
Skins	3.5	Collagen and gelatin Antimicrobial compounds	Atef and Mahdi [15] Caruso et al. [16]

Bones	10–15	Calcium Collagen Lipids	Suresh, Kudrey, and Johny [17] Caruso et al. [16] Coppola et al. [18] Kandyliari et al. [19]

Scales	2	Collagen Chitin and chitosan	Molina-Ramírez et al. [20] Caruso et al. [16]

Head	10–15	Protein hydrolysate Lipids Gelatin	Y. Zhang et al. [21] Prihanto, Nurdiani, and Bagus [22] Kuang and Mohtar [23]

Viscera	12–18	Fish oil Enzymes (trypsin) Probiotic	Caruso et al. [16] Wang et al. [14]

Swim bladder	39.2	Collagen/gelatin	Li et al. [24]

**Table 2:** Nutritional content of fishery by-products.

**Fish**	**By-product**	**Humidity (%)**	**Lipids (%)**	**Proteins (%)**	**Ash (%)**	**Reference**
Nile tilapia (*Oreochromis niloticus* [*O. niloticus*])	Head, frame, and tail	66.50	8.90	5.50	14.6	Torres-León et al. [4]
	Head	67.30	10.90	12.30	9.50	Moraes et al. [52]
	Skin	67.14	1.96	29.08	1.82	Franco et al. [53]

Yellowfin tuna (*Thunnus albacares*)	Skin	76.03	0.24	22.79	4.24|	Sousa et al. [54]
	Head	70.10	7.70	15.10	5.18	D. Oliveira et al. [55]
	Dark muscle	66.80	5.90	ND	8.10	Montiel-montoya et al. [56]

Meagre (*Argyrosomus regius*)	Head	64.0	28.88	40.41	20.95	Kandyliari et al. [19]
	Intestines	73.0	17.09	59.62	4.77	
	Trimmings	63.1	3.00	45.87	49.12	
	Skin	58.4	6.12	75.16	20.24	

Gilthead sea bream (*Sparus aurata*)	Head	57.3	37.08	32.40	18.11	Kandyliari et al. [19]
	Intestines	67.1	43.19	37.23	3.57	
	Trimmings	48.6	5.45	41.85	45.76	
	Skin	53.0	46.39	43.16	6.02	

Rainbow trout (*Oncorhynchus mykiss*)	Viscera	60.26	31.18	12.18	1.33	Polat, Tokur, and Buga [57]
	Head	62.40	6.0	29.0	1.91	Li et al. [41]
	Skin	64.35	1.37	31.66	2.71	Ramezanzade et al. [58]
	Trimming	66.50	44.0	45.10	3.0	Vázquez et al. [59]

Abbreviation: ND, nondefined.

**Table 3:** Antioxidative activity of FPHs from different fish by-products.

**Fish specie**	**By-product**	**Type of assay**	**Objective**	**Outcome**	**Reference**
Rainbow trout	Viscera	*In vitro*	Protein hydrolysates of rainbow trout viscera were subjected to simulated gastrointestinal digestion and intestinal absorption in an *in vitro* assay.	• Protein hydrolysates with more bioavailability. • Peptides could penetrate *in vitro* cells, showing their easy absorption. • They show to be a promising alternative to inhibit the activity of Angiotensin 1-converting enzyme (ACE).	Vásquez et al. [73]

Common carp	Head Skin Skeleton	*In vitro*	Evaluate antioxidant properties of four peptide fractions derived from the collagen hydrolysates	• The four peptides showed to have an excellent antioxidant activity. • Those with > 30 kDa had better foam capacity, foam stability, and emulsifying activity index.	González-Serrano et al. [74]

Salmon Mackerel Herring	Viscera Head Backbone	*In vitro*	Evaluate the antioxidant and cytoprotective capacity of protein hydrolysates from different fish species using cloned human colon adenocarcinoma cells.	• Hydrolysates had the highest cytoprotective effect in adenocarcinoma cells • The best antioxidant activity against ROS.	Taroncher et al. [70]

Monkfish	Head Viscera	*In vitro*	Evaluate the antioxidant activity and digestibility of protein hydrolysates of monkfish heads and viscera.	• Digestibility and antioxidant activity were higher in monkfish viscera protein hydrolysates. • The levels of cadmium in fish viscera were higher than normal values.	Vázquez et al. [75]

Rainbow trout	Skin	*In vitro*	Investigate rainbow trout protein hydrolysates of different molecular weights with anticancer and antioxidant activities.	• Proteins with a molecular weight of <3 kDa had the highest cellular inhibition in HCT-116 (human colorectal carcinoma) cells. • Rainbow trout peptides showed a valuable anticancer activity.	Yaghoubzadeh et al. [71]

Carp	Skin	Mice	Determine the antioxidant ability of carp skin protein hydrolysates to attenuate plasma/serum levels of oxidative stress markers in adult rats.	• FPHs are safe for animals; however, they have a low biological value. • They can be used as food additives with antioxidant properties.	Tkaczewska et al. [76]

Miiuy Croaker	Swim Bladders	Cell culture	Study the protective function of hydrolysate of miiuy croaker (*Miichthys miiuy*) swim bladder on H_2_O_2_-induced oxidative damage to human umbilical vein endothelial cells (HUVECs).	• The sequence Phe-Pro-Tyr-Leu-Arg-His (FPYLRH) could inhibit H_2_O_2_-induced oxidative stress in HUVECs.	Cai et al. [72]

**Table 4:** Antimicrobial activity of FPHs from different fish by-products.

**Fish specie**	**By-product**	**Type of assay**	**Objective**	**Outcome**	**Reference**
Codfish	Fish skin powder	Mice	Evaluate the effects of fish-derived hydrolysates on the gut microbiota.	• Gut microbiota regulated in obese mice, including *Bacteroides*, *Helicobacter*, *Alistipes*, and *Odoribacter*	Axarlis et al. [82]

Herring	Milt	Mice	Evaluate the impact of low doses of herring protein hydrolysates on the development of obesity-related metabolic disorders in mice. The effects on insulin and glucose tolerance and gut microbiota profile were evaluated.	• Mice fed with retentate astaxanthin showed an improved glucose tolerance without weight gain. • Mice presented an abundance of *Lactobacillus* in the gut microbiota.	Durand et al. [83]

Walleye pollock	Skin	Mice	Evaluate the effects of collagen peptides in the regulation of the gut microbiota in mice fed with a high-fat diet.	• The contents of *Lactobacillus*, *Akkermansia*, *Parabacteroides*, and *Odoribacter* spp were increased at a concentration of 800 mg/kg of collagen peptides in mouse food.	Wang et al. [84]

**Table 5:** Antihypertensive activity of FPHs from different fish by-products.

**Fish specie**	**By-product**	**Type of assay**	**Objective**	**Outcome**	**Reference**
Nile tilapia	Skin	*In vivo* Mice	Evaluate the ACE inhibitory effects of Nile tilapia skin protein hydrolysates, stimulating gastrointestinal digestion. *In vivo* and *in vivo* assays were performed.	• *In vitro*: The peptide acted as an ACE inhibitor. • *In vivo*: The mice showed decreased blood pressure after 6 h and a stable digestive process for 4 h.	Chen et al. [89]

Nile tilapia	Skin	*In vitro*	Evaluate the potential ACE inhibitory effect of tilapia skin gelatin hydrolysates and observe its effect on hypertension.	• Key peptides of gelatin hydrolysates did not show competitive inhibitory activity • The peptides displayed strong binding power to ACE sites. • The peptides had the potential to prevent and possibly regulate hypertension.	Ling, Liping, and Yongliang [90]

Lizardfish	Scales	Mice	Characterize the ACE inhibitors from lizardfish scales in hypertensive rats and their potential to be used as a healthy drug against hypertension.	• Gelatin peptides showed an antihypertensive effect in rats at a dosage of 2 g/kg in long-term experiments. • Antihypertensive peptides could be obtained from food sources and eaten without any adverse effects.	Chen et al. [91]

Salmon	Skin Bone Residual meat	*In vitro* Mice	Characterize the ACE inhibitory activity of salmon gelatin in both *in vitro* and *in vivo* assays, stimulating gastrointestinal digestion.	• *In vitro:* Salmon gelatin hydrolysates had more potent antihypertensive activity against the sample without hydrolysis. • *In vivo:* The salmon gelatin showed good antihypertensive properties in rats by oral gavage. • The gelatin hydrolysate provided decreases in arterial blood pressure, systolic and diastolic blood pressure, and heart rate.	Neves et al. [92]

**Table 6:** Anti-inflammatory activity of FPHs from different fish products.

**Fish specie**	**By-product**	**Type of assay**	**Objective**	**Outcome**	**Reference**
*Liza aurata*	Viscera	Mice	Investigate the nephroprotective mechanism effect of *Liza aurata* protein hydrolysates against paracetamol-induced nephrotoxicity and oxidative stress, involving anti-inflammatory activity.	• Protein hydrolysates decreased the leakage of alkaline phosphatase and lactate dehydrogenase. • Considerable protective effect against paracetamol-including kidney damage	Ghorbel et al. [97]

Salmon	Head Backbone	Mice	Evaluate the potential of hydrolyzed fish sidestream proteins to suppress inflammation in mice fed with a high-fat diet.	• Reduction of induced colitis development and proinflammatory cytokine production.	Daskalaki et al. [98]

Anchovy	Viscera	*In vitro* mice	Evaluate the anti-inflammatory potential of anchovy protein hydrolysate by-products in an *in vitro* and *in vivo* model.	• *In vitro:* Significant protection against induced inflammation and decreased the severity of oxidative stress. • *In vivo:* Regulation of the proinflammatory cytokines in the aorta and heart tissues, apart from antioxidant activity.	Giannetto et al. [99]

Sturgeon	Muscle by-product	Mice	Evaluate the therapeutic anti-inflammatory effects and potential mechanism of sturgeon muscle protein hydrolysates on dextran sulfate sodium (DSS)-induced colitis in mice.	• Reduction of the severity of DSS-induced damage and partial restoration of the altered microbiota in mice with colitis.	Gao et al. [100]

Salmon	Bone	*In vitro*	Evaluate the potential anti-inflammatory properties of salmon bone protein hydrolysates on RAW 264.7 macrophage cells.	• Salmon bone protein hydrolysates had the highest anti-inflammatory effects.	Saisavoey et al. [101]

**Table 7:** Antiobesity activity and effects on related diseases of FPHs from different fish by-products.

**Fish specie**	**By-product**	**Type of assay**	**Method**	**Outcome**	**Reference**
Anchovy	Viscera	Mice	Two groups of mice were studied: the first one was fed with a high-fat diet, whereas the second one was fed with a modified diet replacing 10% (w/w) of casein with the same amount of anchovy protein hydrolysates (w/w) for 12 weeks.	• The diet reduced atherosclerotic plaques and lipid accumulation. • Lipid metabolism improved and hepatic enzyme activity reduced.	Abbate et al. [106]

Codfish	Fish skin powder	Mice	Mice were fed with a high-fat diet supplemented with 5% (w/w) of codfish powder and monitored for 9 weeks.	• Insulin resistance suppressed and glucose levels maintained. • The gut microbiota was regulated in obese mice with beneficial bacteria.	Axarlis et al. [82]

Herring	Milt	Mice	Seven groups of mice were fed for 8 weeks with a supplemented diet containing different proportions of herring protein hydrolysate obtained by electrodialysis with ultrafiltration membranes.	• Diets with lower protein content had the best insulin tolerance. • Some diets had an effect on the gut microbiota, owing to their peptide composition and the presence of PUFAs.	Benoit et al. [107]

Walleye pollock	Skin	Mice	Mice were fed with a high-fat diet containing skin collagen peptides (800 mg/kg) to evaluate its antiobesity effects for 4 weeks.	• Collagen peptides showed antiobesity effects in mice, • Serum lipid levels were reduced • Adipose tissue mass was reduced.	Wang et al. [84]

Nile tilapia	Bone Viscera	*In vitro*	Demonstrate if tilapia by-product protein hydrolysates could stimulate hormone secretion and inhibit intestinal dipeptidyl peptidase IV enzyme related to obesity and present in organs, such as the intestine, kidney, or liver.	Promising effects on intestinal hormone secretion and dipeptidyl peptidase IV inhibitory activity were noted.	Theysgeur et al. [108]

**Table 8:** Fatty acid profiles per by-product and species (g/100 g of total fatty acids).

**Fatty acid**	**Gilthead Sea bream (*Sparus aurata)*** ^a^			**Asian sea bass (*Lates calcarifer*) ** ^b^			**Sea bass (*Dicentrarchus labrax*) ** ^c^			**Black Sea anchovy (*Engraulis encrasicolus*) ** ^d^	
	**Head**	**Viscera**	**Skin**	**Head**	**Liver**	**Intestine**	**Head**	**Liver**	**Viscera**	**Head**	**Viscera**
C14:0	2.79	2.64	2.61	0.41	1.29	n.d	2.4	2.2	3.9	7.16	8.33
C14:1	n.d	n.d	n.d	1.31	4.52	5.57	n.d	n.d	n.d	0.04	0.05
C15:0	0.28	0.23	0.23	n.d	n.d	n.d	0.26	0.22	0.32	1.2	1.46
C16:0	13.99	13.27	13.54	47.12	57.84	38.37	16	20	17	20.56	25.81
C16:1*n-*7	5.03	3.68	3.97	n.d	n.d	n.d	4.0	4.0	5.2	n.d	n.d
C17:0	0.54	0.2	0.19	2.38	2.26	n.d	0.49	0.31	0.24	1.96	2.21
C17:1*n-*7	0.43	0.15	0.16	n.d	n.d	n.d	0.0	0.24	0.21	n.d	n.d
C18:0	2.86	3.19	2.87	8.64	4.8	9.89	2.82	5.23	3.14	4.26	6.06
C18:1*n-*9	33.09	35.23	35.41	17.17	20.29	33.37	34	38	32	13.75	13.55
C18:2*n-*6	18.23	19.58	18.87	1.78	1.85	11.56	17	9	13	2.21	1.88
C20:0	0.25	0.3	0.28	n.d	n.d	n.d	0.19	0.18	0.26	0.07	1.41
C18:3*n-*6	0.16	0.21	0.22	n.d	n.d	n.d	0.28	0.27	0.22	0.16	0.1
C20:1*n-*9	1.9	3.06	3.08	n.d	n.d	n.d	2.40	3.0	5	0.64	0.8
C18:3*n-*3	3.86	4.71	4.73	n.d	0.53	n.d	3.69	2	3.3	1.62	1.31
C20:2*n-*6	0.88	0.97	0.88	n.d	n.d	n.d	1.06	0.55	0.73	2.4	1.6
C22:1*n-*9	0.62	0.58	0.6	n.d	n.d	n.d	0.25	0.29	0.51	0.28	0.16
C20:3*n-*3	0.4	0.47	0.5	n.d	n.d	n.d	0.28	0.14	0.23	0.13	0.1
C20:4*n-*6	0.46	0.28	0.28	2.99	n.d	n.d	0.54	0.31	0.35	0.65	0.63
C20:5*n-*3	2.78	1.83	2.03	4.16	2.34	n.d	3.5	3	4.2	10.97	6.93
C24:1*n-*9	0.41	0.49	0.47	n.d	n.d	n.d	0.23	0.27	0.41	0.94	0.82
C22:5*n-*3	2	1.55	1.66	n.d	n.d	n.d	1.20	0.84	1.1	n.d	n.d
C22:6*n-*3	5	3.51	3.98	12.3	n.d	n.d	5.5	4.5	5.3	21.34	18.88
∑SFAs	20.72	19.83	19.72	59.84	64.31	48.25	22	29	24	37.45	46.79
∑MUFAs	44.23	46.05	46.08	18.94	29.61	40.19	43	49	46	22.35	21.31
∑PUFAs	33.77	33.1	33.15	21.23	6.08	11.56	34	22	28	40.2	31.9
*n-*3	14.03	12.06	12.89	16.46	2.86	n.d	13	11	14	34.06	27.22
*n-*6	19.73	21.04	20.26	4.77	1.85	11.56	19	11	14	n.d	n.d
*n-*6/*n-*3	1.41	1.75	1.57	0.29	0.65	n.d	1.37	0.99	1.0	n.d	n.d
PUFA/SFA	1.63	1.67	1.68	0.35	0.09	0.24	1.54	0.76	1.16	1.07	0.68

Abbreviations: n.d, not detected; ∑MUFAs, sum of monosaturated fatty acids; ∑PUFAs, sum of polyunsaturated fatty acids; ∑SFAs, sum of saturated fatty acids.

^a^Pateiro et al. [109].

^b^Chan et al. [110].

^c^Munekata et al. [111].

^d^Gencbay and Turhan [112].

**Table 9:** Summary of the different applications of fish by-product compounds in food systems.

**Bioactive compound**	**Fish**	**By-product**	**Application**	**Effects**	**Reference**
Collagen/gelatin	ND	Skin	Active food packaging films	• Mushroom shelf life was extended up to 10 days more than that of the control samples. • The incorporated films in the foods showed excellent antioxidant activities.	Ahmed, Verma, and Patel [128]
	*Pterygoplichthys pardalis*	Skin	Fresh cheese	• Cheese with collagen skin had higher moisture and protein content than the control samples. • Sensorial properties were not altered, whereas hardness and adhesiveness had slight changes.	Nurubhasha et al. [129]
	*Centrolophus niger*	Skin	Collagen packaging film	• At 5% of pomegranate peel extract, the film with fish skin collagen was effective against *Bacillus subtilis*, *Salmonella typhi*, and *Escherichia coli* (*E. coli*).	Bhuimbar, Bhagwat, and Dandge [130]
	*Mustelus mustelus*	Skin	Collagen bioactive film	• Chitosan helped to strengthen the film and reduce the water solubility. • The film could be used to preserve nutraceutical products.	Slimane and Sadok [131]
	ND	Skin	Buffalo patties	• Fish collagen hydrolysate (FCH) was used as a fat replacer in buffalo patties. • There were no significant differences in the physicochemical and sensory analyses • Formulations with higher FCH had higher protein and ash yet lower moisture content.	Nur Ibrahim et al. [132]

Chitin/Chitosan	*Prochilodus magdalenae*	Scales	Banana starch-based film	• The film inhibited the growth of *E. coli* and *Staphylococcus aureus* cultures.	Molina-Ramírez et al. [20]
	Blue shark	Skin	Red porgy meat	• A 1% chitosan solution applied to red porgy meat improved deterioration indexes. • To improve sensory properties, more studies are necessary.	Liu et al. [133]
	Stripped weakfish	Carcasses and trimmings	Sunflower oil emulsion	• Chitosan particles showed an ACE inhibitory activity and stabilized food emulsion. • The chitosan from carcasses and trimmings can be an alternative as a food emulsion stabilizer.	Oliveira Lima et al. [134]

Fish by-product oil	Sea bass	Trimmings	Fresh pasta	• Fresh pasta with fish by-product oil had stability of fat and an effective protection against oxidation processes. • The cooking time was reduced to 90 s. • EPA and DHA contents were maintained in good quantities during the frozen storage.	Ainsa et al. [135]
	Sardine	Gills and viscera	Wheat flour-based chips	• Improvements in nutritional, antidiabetic, antihyperlipidemic, and histoprotective properties.	Benkhoud et al. [136]
	Cod	Liver	Chicken nuggets	• Fish liver oil did not affect sensory properties of the nuggets • It provided protection against lipid and protein oxidation.	Pérez-Palacios et al. [137]
	ND	ND	Fortified yogurt	• Reduction in acidity, syneresis, and peroxide values. • Samples with fish oils showed higher EPA and DHA contents.	Ghorbanzade et al. [138]

Abbreviation: ND, nondefined.

**Table 10:** Relationship of studies on chemical and enzymatic hydrolysis in fish by-products.

**Fish species**	**By-product**	**Type of hydrolysis**	**Optimal conditions**	**Reference**
*Chemical hydrolysis*				
Persian sturgeon (*Acipenser persicus* [*A. persicus*])	Viscera	Acid treatment	HCl (2 N)	Ovissipour et al. [140]
			pH (3.3)	
			T (85°C)	
			Time (18 h)	
		Alkali treatment	NaOH (2 N)	
			pH (12)	
			T (85°C)	
			Time (18 h)	
Parrotfish (*Chlorurus sordidus*)	Head	Alkali treatment	Minced head: dH2O 1 : 2 (w/v)	Prihanto, Nurdiani, and Bagus [22]
			pH (9)	
			Time (24 h)	
*Pangasianodon hypophthalmus*	Viscera	Acid treatment	HCl (6 N)	Hassan et al. [141]
			pH (2.0)	
			T (110°C)	
			Time (24 h)	

*Enzymatic hydrolysis*				
Salmon (*Salmo salar*)	Frames	Enzymatic	T (60°C)	Idowu et al. [142]
			pH (8.0)	
			Alcalase (3%)	
			Incubation period (180 min)	
Tuna (*Thunnus albacares*)	Viscera	Enzymatic	T (55°C)	Fraterrigo Garofalo et al. [6]
			pH (8.5)	
			Alcalase (1%)	
			Incubation period (120 min)	
Seabream (*Sparus aurata*) and Sea bass (*Dicentrarchus labrax*)	Head Viscera Frames Trimmings	Enzymatic	Solid-to-liquid (S:L) ratio (1 : 1)	Valcarcel, Sanz, and Vasquez [36]
			Agitation (200 RPM)	
			Alcalase (0.5% v/w)	
			Incubation period (3 h)	
Nile tilapia (*O. niloticus)*	Viscera	Enzymatic	T (55.80°C)	Riyadi, Suprayitno, and Sulistiati [143]
			pH (7.9)	
			Alcalase (1.5% (w/v))	
			Incubation period (1.5 h)	

Abbreviation: T, temperature.

**Table 11:** Types of collagens present in fish by-products.

**Types of collagens**	**Source**	**References**
I	Skin Bones Teeth Tendons Organs	Mutalipassi et al. [61] Jafari et al. [150]

II	Cartilages Vitreous body Nucleus pulposus	Jafari et al. [150] Silva et al. [151]

III	Vascular tissues Skin Blood	Alfaro et al. [152]
